# Family language policy and heritage language transmission in Pakistan—the intersection of family dynamics, ethnic identity and cultural practices on language proficiency and maintenance

**DOI:** 10.3389/fpsyg.2025.1560755

**Published:** 2025-03-06

**Authors:** Shameen Fatima, Muhammad Umar Nadeem

**Affiliations:** Department of Mass Communication, School of Social Sciences and Humanities (S3H), National University of Sciences and Technology (NUST), Islamabad, Pakistan

**Keywords:** heritage language transmission, family language policy, language practices, ethnic identity, cultural attachment, bilingualism, multilingualism, family dynamics

## Abstract

Heritage languages play a pivotal role in cultural preservation, ethnic identity attachment and intergenerational continuity. The dynamics of heritage language transmission within family settings warrant comprehensive exploration, specifically in Pakistan, a linguistically and ethnically rich yet under-studied region. This mixed-method study employs qualitative semi-structured interviews (*n* = 7) and quantitative surveys (*n* = 110) to investigate and explore the role of Family Language Practices (FLPs) and policies, family dynamics, cultural and ethnic attachment and attitudes toward heritage language transmission (HLT). The study assumes the theoretical underpinnings of sociocultural and ethnolinguistic identity theory to guide the analysis—utilizing thematic analysis and descriptive and inferential statistics. Findings reveal that the generational gap impacts heritage language maintenance and proficiency, with older generations prioritizing its use and younger generations learning heavily toward culturally dominant languages instead, reasons including societal pressures, personal preferences and the impact of individual experiences. Code-switching emerges as a significant practice amongst the younger generations but also points toward the dilution of heritage languages. Key enablers of heritage language transmission (HLT) were revealed to include multigenerational household structures, cultural practices and positive familial experiences. Additionally, findings reveal the impact of these factors toward the positive and/or negative perception of bilingualism/multilingualism and the perceived importance of heritage language transmission. Strong correlations between language practices, ethnic identity attachment, and family dynamics suggest that intentional, empathetic engagement in FLP can mitigate the challenges posed by modern pressures. Heritage languages are vital for cultural continuity in Pakistan. Policies and practices at the family and societal levels should focus on fostering positive, inclusive experiences with heritage languages to ensure their intergenerational transmission.

## 1 Introduction

Globalization is a rapidly transforming and inevitable part of the modern era (Elewa, 2018; Utomo et al., 2024). In earlier foundational studies, globalization has been attributed to second language acquisition that brings together communities and cultures (Kumaravadivelu and Anonimo, 2008)—later stipulating that cultural globalization, in return, is a critical factor for language learning itself (Tsui and Tollefson, 2007). Language is a fundamental platform for construction of personal and social identity (Ou and Gu, 2021; Tong and Cheung, 2011; Yang and Curdt-Christiansen, 2021) and as such, transmission of language, language maintenance, language shift and language socialization are integral to social interconnectedness. Globalization, while fostering such social interconnectedness, often also forces a certain linguistic homogenization that threatens the survival of more obscure, minor and/or heritage languages (Awal, 2024; Cenoz and Gorter, 2017; Maikanti et al., 2021). The preservation of heritage language emerges as a vital means of sheltering and/or safeguarding cultural and social identities, whether individual/personal or collective.

Amidst this rapid globalization, the factors that both contest and support language maintenance—specifically heritage language maintenance—have advanced exponentially, making it vital to recognize the subtleties that decide upon its success or decline. Heritage languages embody the traditions, histories and identities of communities (Hollebeke et al., 2023; Little, 2020; Montrul, 2023; Tong and Cheung, 2011), acting as critical mediums for transmission of collective memories, norms, values and cultural practices. As globalization takes over the world dynamic, the responsibility for preservation and maintenance of heritage languages essentially falls on families (Danjo, 2021; Little, 2020; Mirvahedi and Hosseini, 2023; Sevinç and Mirvahedi, 2023; Wilson, 2020). As the primary social units, families play a significant role in navigating linguistic demands of the globalized world while also maintaining their cultural ties as demonstrated by studies on cultural continuity and immigrant families (Wang, 2022). However, the ability of a family to successfully transmit, maintain and socialize their heritage language is influenced by several complex intersectional factors including but not limited to socio-economic contexts, culture itself, technology and more.

South Asia presents a particularly compelling space for studying heritage languages and their transmission and maintenance due to the region’s rich and nuanced linguistic range and involved socio-political history (Bose and Jalal, 2022; Driem, 2022; Montanari and Quay, 2019; Wolters, 2018). The region is home to thousands of languages with even more dialects many of which are either endangered or on their way to become endangered due to the dominance of English in a globalized world much like other minority languages across the world (Cenoz and Gorter, 2017; Zhang, 2024). Urban migration intersected with socio-economic benefits of English language proficiency has led to a substantial shift in language use across generations—with younger generations prioritizing dominant languages over their familial heritages and in some cases, completely abandoning them in favor of social acceptance. Institutional policies, such as those in educational institutes, further demean the maintenance of heritage languages by prioritizing English as a mode of instruction to meet global competition and standards. This is further exacerbated in the context of diasporic families, where heritage languages become even more difficult to maintain in host cultures and societies. The pressures of cultural assimilation as well as socio-cultural growth necessitate the learning and use of dominant languages of the host societies which posits greater risk of loss of heritage languages across generations over time (Migliarini and Cioè-Peña, 2024). Family language practices and policies become the core of ensuring language survival in such contexts.

Despite its importance, research on heritage language transmission (HLT) in South Asia remains considerably limited, particularly in socio-politically unstable countries like Pakistan. Existing studies have focused primarily on diaspora communities in Western contexts—for instance, studying the parental attitudes of Chinese immigrants toward their heritage language in the United States of America (Chen et al., 2021), Korean immigrants in New Zealand (Park, 2022), Bangladeshi immigrants in Australia (Chowdhury and Rojas-Lizana, 2021) and even Indian Iranian immigrant families (Mirvahedi and Hosseini, 2023). However, there has been little to no research on the scope heritage language maintenance and transmission when it comes to Pakistan.

This leaves a significant gap in understanding and recognizing the localized contexts of heritage languages within the collectivist culture of the country. There is even lesser focus placed on the endangerment faced by smaller, lesser known and neglected heritage languages spoken only in very specific villages, communities and areas within Pakistan. Addressing this gap is essential for not only managing to spotlight the importance of heritage language preservation and transmission in Pakistan but also for the development of nuanced strategies that provide support to educators, policymakers and other communities and initiatives in fostering retention toward heritage languages.

This paper aims to contribute toward bridging this gap by providing an introductory understanding of heritage language transmission in Pakistan—intersected with familial structures, role of extended family, cultural practices and general perceptions and attitudes toward bilingualism. Drawing on parts of socio-cultural theory and ethno-linguistic identity theory, this study assumes a mixed-method approach to investigate the dynamics of family language policies in multigenerational households within Pakistan. It seeks to uncover the predictors of linguistic proficiency, code-switching and cultural identity while highlighting the potential role of culture and technology in such practices.

## 2 Literature review

### 2.1 Family language policy (or practices)

Family Language Policy (also referred to as FLP or FLPs) provides a robust framework for understanding and recognizing how families make deliberate or unintentional decisions about language practices, language management and socialization within their households (Curdt-Christiansen and Palviainen, 2023; Piller and Gerber, 2021; Schalley and Eisenchlas, 2020). Studies have shown that family language policies, particularly in the case of transnational and/or immigrant families significantly influences language transmission. Factors such as parental approaches (Mak et al., 2023; Said, 2021), immigration practices (Chen et al., 2021; Mak et al., 2023; Mirvahedi and Hosseini, 2023), external environments, societal customs, cultural barriers (Montrul, 2023), education systems (Li and Shen, 2024; Olfert and Schmitz, 2016) both individually and collectively come together to formulate and shape family language policies. Researchers have also stipulated that the role of parents, grandparents and other extended family members as safeguards and gatekeepers (Braun, 2012; Reghiss and Melgani, 2024; Said, 2024) interplays with these factors and emphasizes the significance of intersectional process of intergenerational language teaching and cultural transmission in the process of language learning.

In multigenerational households, where members of different generations often retain varying degrees of not only linguistic expertise but also cultural connection (Purkarthofer, 2020; Sullivan et al., 2021), Family Language Policy becomes a space for compromise and negotiation even if that negotiation is intuitive. The interconnection between explicit policies, for example, dedicated time and space to practice heritage languages—and implicit policies—for example, code-switching during conversations is often fluid and dynamic in nature and not clearly structured. By examining these family language practices, this study aims to uncover the means that encourage or hamper heritage language transmission in diverse family settings within regional contexts of South Asia.

### 2.2 Cultural practices and the role of the extended family

Heritage language transmission operates at the heart of individual agency intersecting with broader socio-economic and cultural structures. Within South Asia in particular, culture and language are deeply entwined, with cultural norms and traditions serving as a backdrop for linguistic maintenance and socialization (Bose and Jalal, 2022; Wolters, 2018). Daily rituals, events, festivals, ceremonies and even religious practices are spaces and opportunities for families to engage in their heritage—reinforcing its emotional, psychological and social relevance and resonance. Grandparents and other extended family members play a vital role in this process. As custodians of cultural heritage, they act as the bridge between generations imparting cultural skills including linguistic competence and values embedded within languages.

In collectivist cultures such as those in South Asia, multigenerational living arrangements are common, even in nuclear living arrangements, the role of extended family remains prevalent due to the collectivist nature of the region (Verma, 1999). In such settings, the inclusive involvement of family members in language learning, language socialization and maintenance foster a sense of shared responsibility. However, the process of maintaining and transmitting languages remains full of challenges in the modern globalized world. Linguistic competence and preferences for other more dominant languages create barriers to influential transmission and preservation of heritage languages. In South Asia, this challenge of language assimilation is intensified by the colonial legacy of English (Gargesh, 2019) as well, which continues to serve as a language of mobility and prestige in socio-economic contexts. These challenges are further made difficult with the advent of social and digital media—often operating in dominant languages only.

### 2.3 Theoretical foundations

This study is grounded in two key theoretical perspectives; socio-cultural theory and ethnolinguistic identity theory, all of which individually and collectively provide a lens to examine the process of heritage language transmission and family language policies. By anchoring the different nuances of these theoretical underpinnings, the study seeks to unpack the diverse dimensions of linguistic development, retention, maintenance and transmission.

#### 2.3.1 Socio-cultural theory

Socio-cultural theory, first posited by Lev Vygotsky in 1978, stipulates that social interactions play a key role in shaping cognitive and linguistic development (Lantolf et al., 2018; Newman, 2018). Vygotsky argues that human development or individual development is a social process impacted and/or mediated by many social and cultural factors. Primarily utilized in fields of psychology and education, sociocultural theory emphasizes the importance of culture on the way individuals act, think and feel (Newman, 2018). While broad, sociocultural theory is pivotal in understanding language learning and transmission across generations—particularly when keeping family structures and community settings in mind. Duff (2019) argues that human existence itself is contextual and that no learning can happen in vacuum. Language learning as part of human existence is a social process, with everyday interactions whether verbal, visual or auditory contributing to language competence and proficiency.

Language is not just a tool of communication but also a socio-cultural artifact that is passed through interaction and carries certain traditions, knowledge of the community and values at large (Park, 2022; Purkarthofer, 2020). For families focused on heritage language transmission, language serves as a repository for cultural heritage as well as a bridge among generations. Sociocultural theory dictates the interdependence between individual learning and the wider social circumstance—highlighting that children effectively acquire and maintain heritage languages when immersed in cultural nuance and robust interactive environments (Hoff, 2006; Roberts et al., 2019; Tizard et al., 1972). This directly correlates to the role of interaction in learning itself. Duff (2019), in her study detailing second language learning mentions multiple levels of interactions that come together to influence second language acquisition including macro level of ideological structure, meso level of communities and micro level of social activity.

Macro levels of ideological structure include all kinds of values and belief systems such as religious values, cultural values, economic values, political values etc. Interactions resulting from these systems both internal and external result in a profound impact on language learning (Duff, 2019). Similarly, meso level of socio-cultural institutions and communities such as families, schools, places of work, places of worship, social organizations etc. directly impact the social identity predictors such as power, attitudes, perceptions and agency that go into learning processes and cognitive development. And lastly, micro level social interaction and activity such as auditory, pictorial, graphical and other forms of interactions become semiotic resources for language learning. Banking on sociocultural theory, Duff (2019) stipulates the dynamic interplay among numerous factors as individuals engage with others in multilingual contexts and in the process learn and use language.

Another key concept that relates positively with these levels of interactions in learning, is the ‘zone of proximal development’ in Vygotsky’s framework in sociocultural theory. It refers to the range of tasks that an individual can learn to perform under the guidance of a more knowledgeable individual (McCafferty, 2002; Irshad et al., 2021). Applying that to the context of interactions and heritage language learning and transmission, parents and grandparents often become facilitators, providing a platform through shared activities, everyday conversations and cultural storytelling as well. These interactions enable younger minds to develop linguistic proficiency while also entwining themselves with their cultural practices and traditions.

Parental attitudes toward these interactions and their direct contribution to language learning become a pivotal part of such practices. Strategies like code-switching amongst the older generation facilitates heritage language retention as well. This is particularly important in many South Asian contexts—including Pakistan, where multigenerational households offer a fertile and informed ground for heritage language transmission. As language is learning through social context and interaction, these households become hubs of cultural learning, where grandparents become central in reinforcing the cultural retention and language learning (Braun, 2012; Said, 2024). This involvement from the older generations becomes a key factor in cultural continuity as well. Purkarthofer (2020) in his work on passing down culture and heritage highlights the importance of such cultural practices that encourage and inspire cultural belonging and emotional bonding.

Sociocultural theory helps underscore the importance of these interactions by arguing that human development and learning is not just a functional process but also deeply symbolic and embeds heritage languages within the matrix of collective identity (Newman, 2018). However, it is important to note here that while this underscores the importance of sociocultural interaction, the modern dynamics are becoming increasingly challenging to traditional practices. Digital communication, excessive modernization, cultural transformation from collectivism to individualism, nuclear family formation and other myriads of modern attributes all posit certain barriers toward heritage language learning and transmission. For example, the increasing reliance on digital media for communication where the dominant languages are balanced between English and other global lingua franca cause significant dilution to the presence and use of heritage languages both online and offline (Duff, 2019). By focusing on these multidimensional attributes, this research aims to explore how individuals with different family structures, levels of interaction, degree of sociocultural impact are impacted when it comes to their language learning and retention, particularly with heritage languages.

#### 2.3.2 Ethnolinguistic identity theory

Aside from sociocultural theory, another key theoretical framework that strengthens the foundation of this study is the ethnolinguistic identity theory contextualized in modern dynamics. First stipulated in 1981, ethnolinguistic identity theory essentially states that the relationship between identity and language is significant and correlated (Singh, 2023). Language socialization and practices encourage or hinder social connectedness (Mohammed, 2022). Individuals and groups connect to specific social groups, communities or identities because of linguistic practices. When considering it in the context of heritage language transmission, it simply highlights the social significance of heritage languages in connecting an individual to their ancestral roots. As previously stated, language learning—or learning of any kind—is a social process that cannot exist in a vacuum (Duff, 2019). Social interactions within communities and societies hinder on language which inherently becomes a valuable framework for preservation of individual and collective identity.

Considering the pretext of this study, i.e., globalization and the interconnectedness of the world, it becomes crucial to understand the space that exists for dominant languages and minority or heritage languages. As such, ethnolinguistic identity theory becomes a key theoretical concept for examining and understanding the processes and/or mechanisms to understand how language serves as a marker of personal and collective identity as well as space for negotiation of these identities (Brosius and Polit, 2011; Ou and Gu, 2021; Park, 2022).

Ethnolinguistic identity theory stipulates that language is not just a means of communication but rather a crucial part of expression of individual agency, identity and community (Giles and Johnson, 1987). Language, at its core, binds people together, creates shared experiences and provides a center for togetherness—facilitating and encouraging a sense of belonging to a community. For example, South Asian immigrants in United Kingdom, United States or other countries come together through shared languages, celebrating their individual identity regardless of regional divide. Communities, support groups and other initiatives for immigrants often hinge entirely on shared language that showcases relatability and sense of community (Edele et al., 2018). Another example of language and identity being interconnected is French individuals almost always preferring to use French as their language of communication, refusing to be dominated by the need to converse in English regardless of the social pressure (Parker, 2019). For immigrants, dispersed communities and/or even indigenous tribes, their ancestral or heritage language also becomes a symbol of cultural loyalty and cultural representation in a world that seeks erasure of their individual identities—often language serving as an act of self-preservation and cultural conservation (Kamau and Motanya, 2024; Nzeaka and Ehondor, 2024) to be passed on in future generations.

Language represents a myriad of conscious and subconscious choices individuals and communities make in reflection of their emotional, psychological and sociocultural values. In the context of heritage languages, heritage language maintenance, transmission and socialization are just as much a part of individual ethnic identity as anything else. Intergenerational transmission of these heritage languages becomes a crucial component of cultural survival and conservation. South Asia, as stated previously, has a rich and nuanced background of diverse heritage languages (Wolters, 2018). In Pakistan, dominant heritage languages such as Punjabi, Sindhi, Siraiki and Pushto are just some of the hundreds of languages spoken in the region. Other lesser known but culturally significant languages include Wakhi, Shina, Burushaski and more—all of which represent individually diverse sets of people and communities. For instance, Punjabi is the heritage language of Punjabis, Sindhi for Sindhis, Shina for a tribe in Hunza, Siraiki for Siraikis. These languages are often the most significant marker of their relevant communities and tribes and as such, become cultural symbols linking generations to generations.

However, it is pertinent to note that despite the cultural-dense environment of Pakistan and the multitude of heritage languages, the institutional support for such languages is very little. However, the status and treatment of these languages on an institutional level remains contested, contributing to a polarized linguistic environment that favors dominant languages such as English. In such a manner, the disconnect between Pakistan’s official language policies and the lived linguistic practices of families creates a significant tension that directly impacts Family Language Policy (FLP). While state policies, as Canagarajah and Ashraf (2013) discuss, promote Urdu and English as primary languages of instruction, this often sidelines heritage languages, creating additional challenges for families attempting to maintain multilingualism at home. The extent to which FLP aligns with or resists these broader institutional policies is crucial to understanding language transmission patterns, language shift, and identity negotiation within Pakistani households. As language policy interacts with both institutional structures and personal decisions, the question of language maintenance becomes not only an issue of national policy but also one of ethnolinguistic identity, shaping how individuals navigate linguistic convergence, divergence, and transmission across generations.

According to Parker (2019), ethnolinguistic identity theory was originally presented to address the concept of who in an ethnic group uses what strategies of language, when and why. Primarily, the concerns were surrounding the explanation of why certain members of a group would accentuate their linguistic differences while others converge toward homogeneity bypassing their linguistic characteristics when conversing with outgroups (Parker, 2019). Over time, the sociopsychological context of language maintenance became an inter-group phenomenon just as much as an individual and personal decision toward letting languages erode or transmitting them ahead. Even today, the cognitive processes relating to social categorization, attitude formation, second language acquisition and more are significant at a macro-level (Giles and Johnson, 1987).

The idea that language maintenance and socialization lie at the heart of a group or community’s vitality directly relates to the ethnolinguistic identity theory. A recent study conducted in Sweden reveals that ethnolinguistic identity and local vitality are closely linked and contribute to explaining the language climate (Lindell et al., 2023). According to Ahmed (2016), ethnic identity maintenance or retention undertaken by small immigrant groups when placed in inter-ethnic contexts has been relatively less explored than the processes that contribute to their adaptation in the host societies. However, he concluded in his study that ethnically minority groups can survive cultural assimilation and maintain their ethnic identity even if the vitality of the group is considerably low on most of the measurable scales (Ahmed, 2016). What gives strength to maintaining their identity is primarily the maintenance of intergenerational heritage language transmission—the use of their ethnic/heritage languages within their homes and neighborhoods is a direct factor. Language, as such, becomes a crucial factor in maintaining ethnic identities, particularly in circumstances and contexts where the ethnic group is a minority.

There are multiple components that influence ethnolinguistic identity formation and maintenance. Daha (2011) conducted an interview-based study on second-generation Iranian Americans revealing that pride in their culture, the physical characteristics of the individuals, the perceived stereotypes etc. all combine to influence ethnic identity maintenance and retention. In addition, she also concluded that the contextual factors that influence such identity retention include community ties, ethnic pride, engagement in cultural practices, family connectedness and cultural beliefs (Daha, 2011)—all of which have been explored in this study in the context of Pakistan. Researchers have also shown that demographic strength, status and institutional support play key roles in how heritage/ethnic languages are strengthened, maintained, encouraged and/or lost over time (Duff, 2019; Lindell et al., 2023; Tizard et al., 1972). In a study done on ethnolinguistic vitality, social identity and intergroup relations in India have further described these factors stipulating that the subjective vitality of any language when it comes to ethnolinguistic contexts depends on these the economic, social, socio-historic or other manners of status of the ethnic groups, the number of speakers or population of the group and the use of the ethnolinguistic groups’ language in different informal and formal spaces (Singh, 2023). In any setup, the groups differ in their relative strength of these three factors, no matter how multidimensional they may be.

Singh’s (2023) research is particularly relevant to the research undertaken because of the similarities in cultural and regional contexts. India and Pakistan share a deeply personal and interconnected history, and the complex multilingualism of India is relatable to that of Pakistan. Groups are difficult to diversify based on dominancy as opposed to the clear distinctions in some monolingual societies of the West (Singh, 2023). In such involved situations, where there is a plethora of regional languages and dialects, the maintenance of languages comes through integration. Different linguistic groups and communities meet each other and move toward multilingualism where minority communities keep their heritage languages while also picking up on the languages of the major groups in specific regions (Singh, 2023). A contextual example of that is someone from the Pukhtoon community within Pakistan—say from Peshawar, a culturally Pukhtoon dominant city migrating to Islamabad, culturally dominated by Punjabi and Urdu-speaking communities would retain their language (i.e., Pushto) while also learning to speak the locally dominant language (i.e., Urdu or Punjabi). In such contexts, ethnolinguistic identities are significantly impacted by social contexts.

As mentioned previously within the context of sociocultural theory, grandparents or the older generations play a vital role in transmission of language and hence, with ethnolinguistic identity theory under consideration, they play an important role in the formation, maintenance and transformation of identities as well (Said, 2024). In cases of culturally collectivist societies like Pakistan, this transmission happens through familial solidarity, practices and familiar decisions. These familial decisions, on an individual level within younger generations, may be diverse and converge away from the norm of the older generations whether through internal values (e.g., personal experiences relevant to heritage languages, individual family dynamics etc.) or external pressures (e.g., social psyche of the environment, the stereotypes, societal pressure of dominant languages etc.) but still remain a deciding factor in the vitality of the ethnolinguistic identity of these younger generations.

Ethnolinguistic identity theory offers a deeply personal perspective on language maintenance, shift and transmission within ethnic groups and provides the lens through which to analyze the findings of this study. As such, ethnolinguistic identity theory is one of the many contexts to consider when exploring heritage language transmission and maintenance, which when considering attitudes and behaviors gives way to another theoretical construct.

## 3 Materials and methods

This study aims to investigate family language policy and heritage language transmission within Pakistan, particularly focusing on family language policy (FLP), family dynamics, ethnic identity and attitudes and perceptions toward multilingualism and heritage as well as dominant languages. A mixed-methods design was employed based on similar studies in research areas surrounding FLP (Kim, 2023), incorporating semi-structured interviews aimed at collecting qualitative data and a quantitative survey questionnaire. The research instruments were designed in a way that facilitates the assessment of intersectional interplay of language use patterns, family dynamics, cultural/ethnic identity and attitudes and perceptions—all underpinned by theoretical constructs of sociocultural theory and ethnolinguistic identity theory. Data analysis combined qualitative thematic analysis using Dedoose software for analyzing interviews and SPSS for statistical evaluation of the survey responses.

### 3.1 Qualitative method: semi-structured interviews

#### 3.1.1 Participants

Following similar studies (Kim, 2023; Mambetaliev, 2023) focused on understanding the nuances associated with heritage language transmission, this study utilizes purposive sampling and employs seven participants in the interview phase—all with diverse backgrounds, diverse household structures, linguistic practices, ethnic heritages and family dynamics. Participants ranged from ages 18 to 35 and represented various ethnic backgrounds including Urdu speaking immigrants, Punjabi, Sindhi, Pashtun, Wakhi etc. ensuring that while the entirety of Pakistan’s hundreds of regional and ethnic languages cannot be represented, the participant group is diverse as such that it provides comprehensive insights.

The study was conducted in Islamabad, Pakistan, a city that serves as a unique linguistic and cultural intersection, attracting individuals from different ethnic backgrounds and provinces. As the capital, Islamabad provides an environment where linguistic diversity is actively negotiated in family and social settings, making it an appropriate site to examine heritage language transmission.

Participants were selected based on specific criteria to align with the study’s focus. Given the purposive sampling approach, the study sought individuals who met few key requirements including (1) they belonged to a multilingual household where heritage language transmission was a lived experience, (2) they represented a range of ethnic and linguistic backgrounds to ensure diversity in heritage language experiences, and (3) they came from different household structures, including nuclear and multigenerational families, to assess how family composition influences FLP. By incorporating participants from varied linguistic and household backgrounds, the study aimed to provide a nuanced understanding of the sociocultural and identity-driven factors shaping heritage language maintenance in Pakistan.

#### 3.1.2 Instrumentation/interview

The interview structure included multiple sets of open-ended questions—divided into nine short sections; demographics detailing personal background and language background, family language practices including language use across generations and within intergenerational language learning, role of extended family and cultural and ethnic identity including heritage and cultural practices and perceptions of bilingualism, challenges in maintenance of heritage languages including parental strategies, future perspectives on language transmission, emotional and psychological impact, cross-cultural adaptions influences and future language outlook. Questions were designed based on existing literature to provide relevance and theoretical foundations—by exploring sociocultural theory talking about the impact of contexts and environments (Lantolf et al., 2018; Newman, 2018) and ethnolinguistic identity theory (Giles and Johnson, 1987; Lindell et al., 2023) relating ethnic identities to linguistic practices detailing questions on attitudes and perceptions.

The interviews lasted between 35 and 60 min, with the shortest interview recorded at 35 min and the longest at 57 min. On average, most interviews fell within the 38–46 min range. Additionally, interviews were conducted with linguistic flexibility, allowing participants to speak in English, Urdu, or their heritage language if they preferred. However, most participants chose to respond in English, with only occasional instances of Urdu usage. In rare cases where heritage languages were spoken, responses were transcribed and translated into English for consistency in analysis.

#### 3.1.3 Process

Interviewees were chosen through purposive sampling to ensure diversity. Each interview was conducted virtually, utilizing Google Meets and its recording feature. Considering the aim of having people from diverse backgrounds, online interviews were found to be more favorable with little regard for distance or geographical boundaries across the country. Participants found them to be easier to engage in as well. All interviews were audio-recorded with consent, taken both verbally before the recording itself and then after, to ensure documentation of consent for each participant if needed. Interviews were transcribed using Tactiq—a transcription tool specializing in providing transcripts, highlights and summaries of online meetings using AI. However, to ensure all transcripts are verbatim for analysis, each of them was matched manually to the audio-recordings. All personally identifiable information was redacted from the transcripts to ensure anonymity and confidentiality.

#### 3.1.4 Data analysis

Thematic analysis was chosen as the method of data interpretation, analyzing the interviews using a six-part framework—starting with familiarization with the cleaned transcripts, generating initial codes from the data, searching for themes, reviewing them, defining them and producing detailed discussions. A cloud-based qualitative data analysis tool called Dedoose was utilized to facilitate the process in managing and coding the data. Code frequencies were generated using Dedoose as well as code co-occurrence insights. All emergent themes were references to existing theoretical foundations of concepts under consideration for this study.

### 3.2 Quantitative method: survey questionnaire

To ensure the findings of the study are not only reliable but helpful in the formation of foundational strategies and help further existing research, a quantitative method utilizing survey questionnaire was also employed in addition to the semi-structured interviews.

#### 3.2.1 Participants

The survey was distributed using networking channels such as WhatsApp and Instagram and utilized a snowball sampling technique. The basis of this study was to provide a foundational understanding of heritage language transmission and family language policies in Pakistan and as such, there were little restrictions to the attributes of the participants. The survey was completed by 110 respondents, including individuals from diverse backgrounds, different household structures and different generational family roles as well. The demographic breakdown and other results are provided in the following sections.

#### 3.2.2 Instrumentation/items

The survey (see Table A1) comprised of six key sections. Demographic information included items aimed at differentiating age groups, gender, primary language, heritage language, ethnicity, household structure and generational family roles. The succeeding four sections detailed four variables—language practices (Lee, 2021; Aguskin, 2023), ethnic identity (Subramaniam and Carolan, 2022), family dynamics (Ong and Ting, 2022), and attitudes and perceptions (Chowdhury and Rojas-Lizana, 2021) with 5, 3, 5, and 5 items each, respectively. The survey utilized Likert scales and multiple-choice questions to capture data on the variables. Each variable/item was informed by existing concepts, frameworks or measures.

#### 3.2.3 Process

The questionnaire was formulated and dispensed online using Google Forms to ensure ease of response, accessibility to data, and participant recruitment. Participants were recruited using networking sites and social media platforms to ensure a broader, diverse demographic reach. Instructions on the survey form ensured the participants knew of their academic participation and confidentiality. All responses were collected anonymously with no mention of any personally identifiable data. The data was cleaned and coded using nominal or ordinal measures where needed and was prepared for statistical analysis.

#### 3.2.4 Data analysis

Statistical analysis was conducted using IBM’s SPSS. Descriptive statistics reported on the demographic items as mentioned above. The basis was to examine the frequency distribution and self-reported measures. Furthermore, inferential statistics were applied to explore the relationship between the other variables outside of demographic items. Reliability tests also confirmed the internal consistency of these variables, with Cronbach’s alpha values exceeding 0.7 for all scales.

## 4 Results

### 4.1 Qualitative findings

This section presents the qualitative findings of our study on FLP and heritage language transmission and maintenance in households within Pakistan. The findings reveal comprehensive insights into four key areas, referenced ahead. Themes emerging from the qualitative data illustrate the complexity and nuances between the intersection of heritage language transmission, family language practices, cultural and ethnic identity, family dynamics and relevant attitudes and perceptions toward bilingualism/multilingualism and heritage languages.

The findings are grouped under thematic headings based on code applications and code co-occurrences analyzed through Dedoose. Seven interviews, as mentioned before, were conducted online. An overview of the general demographic information on the participants is illustrated in Table 1 as follows.

**Table 1:** Demographic overview of interview participants, P1 to P7.

#	Age	Gender	Heritage language	Ethnicity	Household structure
P1	40	Female	Urdu	Urdu-speaking Muhajir	Nuclear family
P2	24	Male	Wakhi	Wakhi Tajik	Multigenerational
P3	22	Female	Burushaski	Burusho	Nuclear, previously multigenerational for 1 year
P4	21	Male	Hindko	Hazara/Hazarvi	Nuclear family, previously multigenerational for 20 years
P5	27	Female	Siraiki/Saraiki	Siraiki	Multigenerational
P6	24	Female	Urdu	Urdu-speaking Muhajir	Multigenerational
P7	23	Male	Punjabi	Punjabi	Multigenerational

The participants represent diverse ethnicities, diverse household dynamics and diverse heritage languages. The research utilized purposive sampling to ensure diversity in the sample. Primarily, individual participants were chosen from the age groups between 18 and 44, mostly those from the generations that could provide insight into both role of the older generations in the interplay of languages as well as provide perspective on the future younger generations and their connection to heritage languages. As illustrated above, the sample includes participants who live in multigenerational households currently, those who did before for diverse periods of time and have now moved to nuclear families and those who have lived in nuclear families only. This aids in the provision of varied perspectives on the family dynamics.

#### 4.1.1 Thematic analysis

Initially, in the process of analyzing the manuscripts, four coding categories were developed—family language practices, ethnic identity attachment, family dynamics, attitudes and perceptions toward heritage languages (Table 2). Each coding category was divided into further sub-codes as follows.

**Table 2:** Key themes emerged in qualitative data.

#	Key theme	Sub-themes
1	Family Language Policies and Dynamics	Contextual Use of Language
		Intergenerational Language Use
		Parental Strategies
		Emotional Connection
		Family Traditions
		Influence of Extended Family
2	Language Learning and Proficiency	Competence and Proficiency across Generations
		Code-Switching
		Cultural Practices and Assimilation
3	Ethnic Identity Attachment and Importance	Language and Ethnic Identity
		Perceived Importance of Heritage Language Transmission
4	Attitudes and Perceptions	Positive Perception of Bilingualism/Multilingualism
		Benefits of Bilingualism/Multilingualism
		Impact of Personal Experiences
		Perceived Challenges in HLT

The codes were created keeping the four key variables in mind as well as the theoretical foundations as illustrated previously. After the initial coding of the interview transcripts, Dedoose was used to understand the frequency counts and the co-occurrences of the codes to refine the findings—identifying patterns and broader constructs to combine overlapping themes. Four broad themes have emerged from the interview data, with deep insights pointing toward the complex interconnectedness of the different variables involved in heritage language transmission.

##### 4.1.1.1 Theme 1: family language policies and dynamics

The first theme highlights how family language practices and policies influence the use of heritage languages as well as the ways in which family dynamics impact competence and individual connection with heritage languages. Families employ both conscious and unconscious language policies to navigate intergenerational communication, contextual use of heritage languages, language connections and more.

Participants reported that the use of both heritage languages as well as primary languages varied based on specific contexts. For instance, heritage languages were more notably used during family events, gatherings, cultural ceremonies and rituals while conversely, in professional or educational settings, dominant languages overshadow heritage languages. This divergence reflects the duality of languages and the ways they are utilized in terms of contextual setting.

For example:

**P3:** *“Yes, when there are family, extended family, gatherings, including marriages, or any sort of ceremonies…these are the events in which we prefer to, like, talk in our native language and all the ceremonies and all the rituals are also performed in our own language.”*

**P3:** *“Since English is the language in all our offices and all of the workplaces, it’s the workplace requirement these days. It’s the requirement of all the educational institutes as well. There are all kinds of pressures to learn and maintain English as a dominant language. These pressures force us to leave our heritage languages behind and that keeps on increasing as well, the pressures I mean.”*

A significant subtheme observed under language practices is also the varying use of heritage language among different generations. Participants reported that heritage languages are often a preference of the older generations more so than the younger ones. Older generations, particularly grandparents and great grandparents, often converse exclusively in their heritage languages and frown upon the use of other languages within the household. Younger generations, in contrast, were reported to either exhibit the use of a mix of primary and heritage languages—frequently code-switching—or favoring culturally dominant languages (e.g., English or Urdu depending on the environment) altogether.

For example:

**P6:** *“Yes definitely, a 100% when it comes to my grandparents and my parents since they are Urdu speaking as well as their education. Like my mother’s education has been in Urdu literature…my grandfather was a poet…they have clarity in their speech. But when it comes to the younger generation such as my siblings or my cousins, the generation that comes after me. Maybe they are not quite proficient. They pollute the language and like to use the words of Urdu and English together. They couple it, so it’s more like a cross-reading of the two languages and there is influence of the English accent, even in Urdu language.”*

Respondents reported that parental involvement and strategies have played an active role in encouraging the use of heritage languages, explaining that their proficiency often comes because of their parents conscious or unconscious efforts steering them closer to their respective heritage languages. These strategies included dedicating language practice, cultural rituals, storytelling, engaging in literature and more. However, some participants also acknowledged the challenges in maintaining such practices in contemporary times.

For example:

**P1:** *“My father used to take me and my siblings to the local library, and he used to force us that everyone is allowed to take two books home and one of them particularly has to be in Urdu. So, I remember that from childhood. Like when I started reading it, probably at four or five years of age, I used to read one Urdu book and one English book per week, that I think made my vocabulary better than my children.”*

Heritage language was reported to be a source of intense emotional experiences as well—both negative as well as positive. Participants often associated their own perception and connection to the language based on the kind of emotional experiences they had with their language. While there was a mix of feelings reported by the participants, it was clear that participants that had a primarily positive emotional experience with their family when it came to the heritage language would prefer to associate with it more than the participants that had a negative experience.

For example:

**P5:** *“… with my naana naani (maternal grandparents), I used to speak Siraiki and they were happy with that and I genuinely connected with them at many levels. I would say because of that language, because they were always, you know, giving me the right amount of attention when I was speaking. And as a kid who doesn't want to attention, right? It was a point of connectedness and warmth and made Siraiki very important to me…”*

Much like parental strategies, family traditions and practices were also reported to be pivotal in heritage language use and transmission. Participants highlighted that family traditions such as festivals, religious ceremonies and even culinary practices required the use of their heritage languages more so than any other dominant language—ensuring that in some ways, the heritage languages remained relevant. These family traditions carry a lot of weight when considering the fact that most families confine heritage languages to their homes only and prefer other languages outside of them.

For example:

**P1:** *“…these and also some Islamic events like if we have a milaad at our home, we cannot engage in any other language, than our heritage language. So even if everyone sitting there does not understand Urdu even then the person who has come to deliver the lecture or deliver the milad, so what we do is we engage in certain lecture, certain Islamic lectures. So even then, if there is a person who has come to give the lecture, he will not even use a single English word in that particular lecture. People are understanding or not, he does not care.”*

Respondents stipulated extended families to be one of the main contributors toward learning and maintaining their heritage languages, particularly grandparents, aunts, uncles and even cousins. While family gatherings became important spaces for reinforced use of heritage language, extended family members became custodians of heritage languages and facilitating the process of learning and acquiring the language. Grandparents were reported to be the most valued when it comes to language maintenance.

For example:

**P4:** *“I think the 80% of the role was them [grandparents] 80 to 90% of the role was them, because even though they knew how to speak Urdu but they prefer to speak in Hindko and we simultaneously used to speak it with them as well. So in the conversation exchanges we learnt the language from [grandparents].”*

##### 4.1.1.2 Theme 2: language learning and proficiency

The second broad theme observed focuses on the diverse levels of language proficiency and competency across generations, the language practices and the different perspectives on heritage languages themselves.

Participants reported significant differences across generations in the modern world—their heritage language proficiency, competence and preference. While it was clear that the older generations were fluent and highly proficient in their heritage languages, it was stipulated that the younger generation often demonstrated limited vocabulary, struggled with syntax and grammar and reflected a general lack of language expertise. Furthermore, it was also reported that the younger generation often preferred the culturally dominant languages instead of their own heritage language.

For example:

**P3:** *“Yes, there’s a huge difference because there are lots of people in my family, the, especially the younger generation, they cannot understand anything in the native language and even though they find it really difficult to connect with these all these rituals and ceremonies where our heritage language is being practiced. So yes, there’s a huge gap.”*

Another sub-category under the broader theme of language proficiency is the code-switching behaviors differing across different generations. Code-switching emerged as a notable practice amongst the younger generations—participants described how the younger generations often alternated between their heritage languages and the culturally dominant language, partly because of limited expertise in heritage languages and partly to navigate social and professional spaces. The importance of preserving the purity of their heritage language remained relatively low in the younger generations, impacted heavily by the changing times.

For example:

**P2:** *“My younger sister when she speaks Wakhi and mixes other words in it. She says a sentence in Wakhi but uses English also in it.”*

Relevant to code-switching behaviors as well as the lower proficiency rate of heritage languages amongst the younger generations is also cultural assimilation and cultural practices. Participants reported that cultural assimilation in environments outside of their own ethnic communities acted as barriers to language retention and maintenance, lowering language competence and proficiency. On the other hand, practices and rituals within their communities strengthened language maintenance and retention.

For example:

**P3:** *“Few words have been added to Wakhi, for example, a couple of years back, someone pointed out to me that some of the words I use daily in Wakhi are not Wakhi words. They are Burushaski and it has been normalized that they were Wakhi and we use them daily. That is basically an influence, a cross-cultural influence.”*

##### 4.1.1.3 Theme 3: ethnic identity attachment and importance

Adding to the conceptual foundation of this study, of ethnolinguistic identity theory, this theme explores the connection between ethnic identity and heritage languages, placing an emphasis on the perceived importance of heritage languages in the respondents’ lives.

Respondents reported that heritage languages have been a vital marker of their ethnic and cultural identities regardless of their level of language proficiency. Expressing a sense of connection and pride in their linguistic heritage, participants viewed language as a huge part of preserving culture and ethnicity and fostering a certain sense of belonging to their community. They expressed that it is plausible that their ethnic identities might not remain intact if their heritage and ethnic languages are lost or phased out.

For example:

**P2:** *“We have basically lost all other parts of our culture to modernisation. The language is the only thing that remains. We live in Islamabad, so we have to maintain the daily life according to that. We do have some parts of our culture, we do cook traditional foods occasionally. But not daily, of course. So that is also somehow lost. But the only thing that remains is the language that we use daily. That is the only thing that has been with us. So if the language is lost, then I guess the culture is lost.”*

Most, if not all, participants emphasized the importance of heritage language transmission to future generations, suggesting the criticality of maintaining languages as means of maintaining cultural identities. Participants noted that because of their feeling of connectedness to their community as well as the potential loss of identity, they believe transmitting their language to future generations is vital, even if difficult.

For example:

**P3:** *For me personally, my heritage language does have a lot of importance in my life. I will prefer to communicate with my parents, with my siblings with my younger cousins, and everybody in my own heritage language. Because, for me, personally, it plays a huge role in my life. I feel more connected whenever I have a chance to speak, even with my friends from back at home, in my own language.”*

##### 4.1.1.4 Theme 4: attitudes and perceptions

The final theme emerging from the data is the participants’ attitudes and perspectives toward bilingualism—both positive and negative. Their perception of the challenges, efforts, rewards etc. associated with heritage language transmission and maintenance are varied and represent their own individual experiences as well.

Participants highlighted the social, cognitive, familial and professional benefits of being bilingual or multilingual—regardless of whether their heritage languages were part of being bilingual or not. They believed that retaining multiple languages provided them and their families greater space for opportunities. Maintaining their heritage language alongside a dominant language meant they could navigate both personal and professional spaces with ease.

For example:

**P5:** *“Okay, my general perspective on this, is that being a bilingual is helping me at least, if I talk about just myself right now, right? So it’s helping me to express myself, more confidently. For example, if I’m talking again to my, for example, household staff, I will feel confident in talking to them and I generally feel that I’m bonding with them with all the grace and I’m building a connection with them because I really love my house help more than even my parents or siblings. So they are so kind to me and I am so kind to them. And I express that in Siraiki because that is the language that connects us and they feel more seen in it.”*

Outside of a positive perspective toward bilingualism or multilingualism, participants also supported that the ability to communicate in multiple languages meant being able to connect with different communities and different kinds of people. It enhanced confidence, connection and provided specific benefits socially as well.

For example:

**P3:** *“Yes, you might be able to connect with more than one type of people, if you can speak more than one language.”*

As previously mentioned in Theme 1, emotional connections are a huge contributor toward heritage language use and maintenance. Similarly, personal experiences with heritage languages have also been reported to contribute significantly toward attitudes and perceptions of heritage languages, their use and transmission. Participants with positive experiences associated to their heritage languages leaned more toward trying to preserve it, while those with negative experiences and emotions associated to their heritage languages were less caring toward the idea of maintaining their language.

For example:

**P5:** *“Because, um, when I was a child, when I was hit by my mother, the exchange was in Siraiki. And it continued and became a language of hatred for me. After that, there have remained stages in my life where whenever I was belittled or discouraged, it was always in Siraiki. Whenever there was violence, it was always Siraiki. So benefits, I don’t think there are many.”*

Attitudes and perceptions toward heritage language maintenance also included perceived challenges and barriers. As such, participants acknowledged that, for various reasons, there are educational, societal, professional and other kinds of pressures associated with heritage language maintenance and transmission. Dominant languages, like English, are prioritized in educational and professional settings instead of heritage languages, leaving little to no room for support toward these heritage languages. Similarly, families have now been compelled to focus on teaching English to the younger generations to ensure their mobility in the society—stripping them off their heritage languages. This has caused the younger generation to also exhibit disinterest and/or resistance toward learning their heritage languages, thinking of it as less important in their lives.

For example:

**P7:** *“Yeah, exactly. I think Punjabi is neither considered a professional language at all. Nor it is associated with people who are very professional. So I think when it comes to societal pressure that was again a very big, contributing factors in me, not trying to learn Punjabi, or speak at it, like, speak it very commonly because it wasn’t considered professional or it wasn’t being taught in our system as such.”*

### 4.2 Quantitative findings

To validate the findings of the interviews, this section presents the quantitative findings of our study on FLP and heritage language transmission and maintenance in households within Pakistan. The results are organized by descriptive statistics and inferential statistics to provide an understanding of the comprehensive data.

#### 4.2.1 Descriptive statistics

The study surveyed 110 voluntary participants with diverse representations across multiple demographic items including age, gender, household structures, generational roles, primary and heritage languages and ethnicity. Table 3 demonstrates the representation of age and gender among the participants. Participants aged 25–34 formed the largest group at 47.3%, following closed by the group aged 18–24 at 37.3%. Older groups were generally less represented in the respondents with only 9.1% in the age group 35–44, 4.5% in the age group 45–55 and an even lesser 1.8% in the age group over 65. Furthermore, the respondents were primarily female with 59.1% representation with males at 40.0% and a mere 0.9% non-binary representation.

**Table 3:** Descriptive statistics, age and gender.

		Frequency	Percentage
Age	18–24	41	37.3
	25–34	52	47.3
	35–44	10	9.1
	45–55	5	4.5
	65+	2	1.8
Gender	Male	44	40.0
	Female	65	59.1
	Non-Binary	1	0.9

On the other hand, household structures were diverse. As illustrated in Table 4, 38.2% of the participants currently live in multigenerational households. 50% have previously lived in multigenerational households and only a mere 11.8% have never lived in such settings. Generational family roles also provided a varied perspective. 69.1% identified as part of the younger generation (for example, children or grandchildren), 22.7% identified as the parent generation and only 8.2% identified as the elder generation which is also reflected in the age groups of the participants.

**Table 4:** Descriptive statistics, household structure and family roles.

	Frequency	Percentage
Household structure		
I live in a multigenerational household currently	42	38.2
I have lived in a multigenerational household before, not at present	55	50.0
I have never lived in a multigenerational household	13	11.8
Generational family role		
Elder generation (e.g., grandparents or similar)	9	8.2
Parent generation (e.g., parent, guardian, or similar)	25	22.7
Younger generation (e.g., child, grandchild, or similar)	76	69.1

Furthermore, the diversity of Pakistan’s ethnic and linguistic divisions is demonstrated clearly in Table 5. While there are diverse ethnic groups across the country, the sample only demonstrates a few of the more prominent ones. Punjabis are the predominant representation in the sample with 48.2% with Siraikis and Pashtuns at similar representation with 10.9 and 10.0%, respectively. The other ethnic groups variate between 0.9 and 12% at maximum. It is important to note here that Urdu refers to Urdu-speaking muhajirs—one of the ethnic groups migrating from India to Pakistan during the partition of the Sub-continent.

**Table 5:** Descriptive statistics, ethnicity, primary and heritage language.

Ethnicity			Primary language			Heritage language		
Urdu	8	7.3	Brohi	1	0.9	Balochi	1	0.9
Punjabi	53	48.2	Brushaski	1	0.9	Burushaski	4	3.6
Saraiki	12	10.9	English	10	9.1	Dogri	1	0.9
Pashtun	11	10.0	Hinko	1	0.9	Hindko	6	5.5
Muhajir	9	8.2	Kashmiri	2	1.8	Hyderabadi	1	0.9
Gilgiti	1	0.9	Pastho	3	2.7	Kashmiri	1	0.9
Pothohari	2	1.8	Punjabi	5	4.5	Pashto	8	7.3
Hazarvi	5	4.5	Urdu	86	78.2	Potohari	5	4.5
Burushaski	4	3.6	Wakhi	1	0.9	Punjabi	49	44.5
Kashmiri	3	2.7				Saraiki	9	8.2
Wakhi	1	0.9				Shina	1	0.9
Brahui	1	0.9				Siraiki	3	2.7
						Urdu	20	18.2
						Wakhi	1	0.9

Here, primary language (also referred to as the dominant language in this study) represents the language most spoken by the participants, the language they are most fluent in and most comfortable speaking. It is important to note that Urdu is the overwhelming majority when it comes to primary language of the participants with 78.2% of the respondents using Urdu as their most frequently spoken language. It is also the official language of Pakistan and hence, the relative importance in professional and personal settings. However, second to Urdu is not any of the heritage languages but rather, English with 9.1%.

Heritage language here, refers to the language that is connected to a person’s cultural or ancestral roots, and is often different from the dominant language in their society or region. The predominant heritage language as demonstrated by the data is Punjabi, inherently representing much of the ethnic group in Pakistan. Urdu-speaking muhajirs consider Urdu their heritage language as well and represent 18.2% of the group’s self-reported heritage language. Other heritage languages include Siraiki, Pushto, Hindko, Kashmiri, Burushaski, Balochi, Sindhi, Wakhi, Potohari, Hyderabadi, Dogri and Shina. Here, Siraiki and Saraiki refer to the same language. The rich heritage and cultural diversity of Pakistan cannot be fully encapsulated in one survey. However, the different languages provide just the tip of the overall range of languages in the country.

It is pertinent to note that for some participants, the primary and heritage languages are the same. They not only speak their respective heritage languages but rather also consider these heritage languages their dominant languages in terms of comfort, fluency and frequency of use.

Language practices were measured across five items. Self-reported primary language competence, self-reported heritage language competence, language use in intergenerational communication, language use in different contexts and code-switching frequency. The sample revealed notable trends (see Table 6) in the use of primary/dominant and heritage language use. Self-reported primary languages indicated relatively high proficiency (*M* = 3.97, SD = 0.983) while heritage language competence was lower (*M* = 2.99, SD = 1.144). Communication across generations relied more on dominant/primary languages as indicated by a mean score of 1.84 (SD = 0.687). Additionally, contextual use of heritage languages scored a little higher at 2.80 (SD = 1.200). On the other hand, code-switching frequency (*M* = 2.75, SD = 1.259) indicated moderate adaptability in the navigation of multiple linguistic environments or spaces.

**Table 6:** Descriptive statistics, key variables.

Variables/items	Min	Max	Mean	S.D.
Language practices				
Self-reported Primary Language Competence	1	5	3.97	0.983
Self-reported Heritage Language Competence	1	5	2.99	1.144
Language Use in Intergenerational Communication	1	4	1.84	0.687
Language Use in Different Contexts	1	5	2.80	1.200
Code-Switching Frequency	1	5	2.75	1.259
Ethnic identity				
Ethnic Identity Scale/Ethnic Identity Attachment	0	1	0.72	0.336
Cultural Identity and Language Socialization/Participation in Cultural Practices	1	5	3.54	1.232
Perceived Role of Heritage Language in Cultural Identity	1	5	3.89	1.120
Family dynamics				
Self-Reported Family Language Influence	1	5	3.81	1.062
Language Practices of Grandparents	1	5	4.19	1.071
Role of Extended Family	1	5	3.17	1.270
Extended Family’s Influence on Language Decisions	1	3	2.03	0.697
Parental Strategies in Language Transmission	0	1	0.40	0.239
Attitudes and perceptions				
Self-reported Importance of Heritage Language Maintenance	1	5	3.49	1.262
Self-reported Challenges of Heritage Language Maintenance	1	5	3.08	1.402
Preferred Language for Household Communication	1	3	2.06	0.951
Attitudes Toward Bilingualism	1	5	3.45	1.020
Perceived Effort and Rewards	1	5	3.45	1.254

The Ethnic Identity measure (see Table 6) indicated strong attachment to participants’ ethnic identity (*M* = 0.72, SD = 0.336). Cultural participation highlighted the participants’ connection to their heritage through engagement in traditional practices (*M* = 3.54, SD = 1.232). Additionally, and notably, the role of heritage languages in ethnic identity was rated highly (*M* = 3.89, SD = 1.120).

Family dynamics played a significant role in giving shape to linguistic practices (see Table 6). The self-reported influence of family language choices was rated at 3.81 (SD = 1.062) with the practices of grandparents scoring the highest (*M* = 4.19, SD = 1.071). Notably, the role of extended family appeared to be moderate (*M* = 3.17, SD = 1.270) while the direct influence of extended family on language decisions was relatively lower (*M* = 2.03, SD = 0.697). Parental strategies for heritage language transmission were relatively on the rarer side, reflected in the score of 0.40 (SD = 0.239)—highlighting challenges in intentional language transmission.

Participants expressed a strong belief in the importance of heritage language maintenance (*M* = 3.49, SD = 1.262), though, self-reported challenges of maintaining these languages were ample (*M* = 3.08, SD = 1.402). Furthermore, respondents leaned toward heritage languages when it came to their preferred household communication (see Table 6). Bilingualism was also highly valued (*M* = 3.45, SD = 1.020). Participants also recognized the effort and rewards of heritage language maintenance (*M* = 3.45, SD = 1.254).

#### 4.2.2 Inferential statistics

This section presents the inferential statistical analysis of the correlations between this study’s key variables: Language Practices, Ethnic Identity, Family Dynamics and Attitudes and Perceptions (see Table 7). To explore these relationships between key variables, Pearson’s correlation analysis was conducted. The findings from the correlation matrix (see Table 7) reveal significant and meaningful relationships, highlighting the interplay between linguistic, cultural, and familial factors in heritage language transmission.

**Table 7:** Inferential statistics for key variables.

Variables	1	2	3	4
Language Practices	**1**			
Ethnic Identity	0.628^**^	**1**		
Attitudes and Perceptions	0.365^**^	0.481^**^	**1**	
Family Dynamics	0.610^**^	0.632^**^	0.451^**^	**1**

The ‘**’ represents *p*-value <0.01 demonstrating the statistical significance of the correlation and underscores the reliability of the relationship. The diagonal values (1s) indicate that each variable is perfectly correlated with itself, as expected in a correlation matrix.

A strong positive correlation exists between Language Practices and Ethnic Identity (*r* = 0.628) illustrating higher levels of engaging in heritage language use and practice is often associated directly with stronger ethnic identity and ethnic attachment. The *p*-value (*p* < 0.01) confirms this correlation is statistically significant. A strong positive correlation also exists between Language Practices and Family Dynamics (*r* = 0.610), indicating that family environments play a key role in influencing language practices. The statistical significance (*p* < 0.01) underscores the reliability of this relationship. A moderate positive correlation is observed between Language Practices and Attitudes and Perceptions (*r* = 0.365), advocating that those who positively engage in heritage language practices tend to have more positive attitudes and perceptions about heritage language maintenance as well. The *p*-value (*p* < 0.01) indicates that this correlation is also statistically significant.

A moderate positive correlation is also observed between Ethnic Identity and Attitudes and Perceptions (*r* = 0.481), suggesting that ethnic identity influences attitudes toward heritage language maintenance. The *p*-value (*p* < 0.01) confirms statistical significance. On the other hand, a strong positive correlation is observed between Ethnic Identity and Family Dynamics (*r* = 0.632) the intersection of both familial and cultural influences on language transmission. The *p*-value (*p* < 0.01) confirms statistical significance. Furthermore, a moderate positive correlation between Family Dynamics and Attitudes and Perceptions (*r* = 0.451) suggests that positive family circumstances influence the attitudes toward heritage language maintenance. The significance level (*p* < 0.01) supports this finding.

## 5 Discussion

The findings of this study provide comprehensive and nuanced insights into the interplay between family language policies and heritage language transmission in Pakistan. By integrating both qualitative themes and quantitative patterns, we have explored the intersectional connection of multiple variables—language practices, ethnic identity, family dynamics and attitudes and perceptions. This section synthesizes these findings and contextualizes them within the theoretical underpinnings of ethnolinguistic identity theory and sociocultural theory.

First and foremost, the findings highlight the notable differences in proficiency, competence and heritage language use amongst the different generations. Older generations, often the custodians of languages as demonstrated by existing literature (Said, 2024), are instrumental in contributing to linguistic heritage. Findings reveal that these older generations prioritize their ethnic and heritage languages over other culturally dominant languages. Their direct influence fosters intergenerational language maintenance and learning—whether through storytelling, rituals, cultural practices or individual choices. Both qualitative and quantitative findings underscore the importance of grandparents, or the older generations in heritage language maintenance and validate existing literature on the matter (Said, 2024; Braun, 2012). On the other hand, it has been revealed that younger generations prefer dominant languages such as English—partly because of personal agency and choice and partly because of societal or professional pressures contributing to the sidelining of heritage languages.

This is further enunciated by the prevalence of code-switching behaviors amongst the younger generations and their navigation of multilingual spaces with a mix of both heritage and primary/dominant languages. Findings reveal that while this code-switching behavior facilitates bilingualism, it undermines the emotional connection of the younger generation toward their heritage and culture and dilutes language purity as well. The quantitative findings further validated the strong correlation between language and ethnic identity as demonstrated by existing studies (Lindell et al., 2023; Giles and Johnson, 1987; Singh, 2023), stipulating that the stronger the attachment to the language or language use, the stronger the ethnic identity attachment as well. This implies that the younger generation does not have the same level of attachment to their ethnic heritage as the older generation.

In addition, the role of family dynamics, family traditions and family roles emerge as pivotal in the maintenance and transmission of heritage languages as well, corroborated through quantitative as well as qualitative findings. It is revealed that family practices and gatherings, family dynamics such as multigenerational households, involvement of extended family etc., all come together to impact language use and language maintenance, by extension, language proficiency as well. A strong positive correlation between language practices and family dynamics points toward the interconnectedness of family and language competence in culturally collectivist societies like Pakistan. Existing research speaks at length about the role of parents, families and the elders in encouraging language use and preservation (Ong and Ting, 2022; Lee, 2021; Chowdhury and Rojas-Lizana, 2021), which has been further endorsed by the findings of this study.

It is important to note here that participants varied in their proficiency of heritage languages and general linguistic competence—heavily influenced by environments and experiences. Sociocultural theory dictates that social contexts and circumstances influence linguistic development (Newman, 2018; Lantolf et al., 2018), which attains another endorsement through the findings of this study. Individuals with social support, positive personal and social experiences and positive family involvement in the process of language learning have been reported to have a better relationship with their heritage languages as well as being more proficient. Language practices having a moderate but considerably important correlation with attitudes and perception further underscores the importance of social contexts and experiences.

Another key construct validated through the findings of the study is the theoretical foundation of ethnolinguistic identity theory which correlates language with ethnic identity and accentuates the importance of this intersection (Singh, 2023). Both qualitative themes, particularly centering around strong ethnic attachments and language preferences, as well as the quantitative correlation between Language Practices and Ethnic Identity indicate that individuals find themselves closely connected to their ethnic roots and heritage identities through their heritage languages. Additionally, language not only serves as a medium of communication but also a touchpoint for genuine connection, closeness and a sense of belonging. Studies detailing the different applications and contexts of ethnolinguistic identity theory and sociocultural theory provide a sound basis for these findings.

The level of heritage language competence as well as language and identity attachment are hindered on multiple interconnected variables and constructs as discussed in the theoretical foundations as well as demonstrated in the findings—positive experiences with languages, family interactions (Lee, 2021), cultural practices (Kamau and Motanya, 2024), parental strategies (Roberts et al., 2019), to name a few. The interconnection of these variables is multifaceted as well.

For example, the findings demonstrate a strong correlation between ethnic identity attachment and family dynamics while also demonstrating a strong correlation between ethnic identity and language practices, all variables being intersectional and contributing to the overall preference, competence, proficiency and agency surrounding heritage language use and maintenance. Furthermore, the discussion becomes even more nuanced when considering the inclusion of attitudes and perceptions surrounding heritage languages and bilingualism. Keeping in mind that social constructs and surroundings impact language learning, among other things, attitudes and perceptions are deeply influenced by these surroundings as well.

Individuals with positive experiences with their heritage languages are often more conducive toward heritage language transmission and maintenance and have a generally more positive outlook on bilingualism. Participants expressed positive attitudes toward bilingualism and recognized the social, professional and cognitive benefits of leaning multiple languages. This also aligns with existing literature on advantages and benefits of multilingualism (Purkarthofer, 2020; Montanari and Quay, 2019). However, findings also talked at length about the challenges associated with heritage language maintenance in the modern world, recognizing that preserving languages is not straightforward and often involves multiple factors—both internal and external pressures. Interestingly, these challenges are mitigated with positive experiences within families and households. Attitudes and perceptions are also impacted by family dynamics and language practices in some ways—positive reinforcement and encouraging experiences with heritage language often result in positive attitudes toward heritage language maintenance and transmission.

These findings accentuate the need for empathetic, intentional and informed family language policies that would help heritage language maintenance and transmission in an involved manner. On the theoretical side, the results of this study support the assertations of socio-cultural theory—that language learning is embedded within social constructs and is informed by interactions within families, with cultural practices and more. Cultural continuity comes from these interactions and social variables. Similarly, the study also supports ethnolinguistic identity theory validating that participants who express strong attachment to their ethnic identity consider language a marker for these associations.

Quantitative correlations also demonstrate the interconnections between the multiple variables—concluding that strong family support and cultural participation enhances heritage language competence and attitudes toward its maintenance while weak or negative family involvement correlates with diminished ethnic attachment and lower preferences for heritage language transmission. Between these interconnections, the impact of cultural practices, parental strategies and other relevant factors is emphasized and validated.

## 6 Conclusion

Grounded in socio-cultural theory and ethnolinguistic identity theory, this study sheds light on the involvement of family language practices in heritage language maintenance intersecting with cultural practices, ethnic identity attachment and family dynamics. Key findings include:

- Older generations play a key role in transmitting and maintaining heritage languages through rituals, cultural practices and storytelling.
- Multigenerational family structures provide ideal spaces for conducive language learning, as far as it is a positive emotional experience.
- A clear generational gap exists in heritage language proficiency, with younger generations favoring dominant languages like English or Urdu.
- Code-switching among younger individuals reflects flexibility and adaptation but risks diluting linguistic heritage.
- Heritage languages are vital markers of ethnic identity, fostering cultural pride and belonging.
- Positive emotional experiences enhance attachment to heritage languages, while negative experiences discourage their use.
- Participants value bilingualism for its cognitive, social, and professional benefits, but societal pressures prioritize dominant languages over heritage languages.

This study is limited to Pakistan, one small part of South Asia and by extension, an even smaller part of the world. This does not account for linguistic or ethnic variations across South Asia or even diasporic contexts within the continent. Furthermore, Pakistan itself has hundreds of linguistic variations and a multitude of ethnic diversity. While efforts were made to make sure the sample was diverse, the vast linguistic space of Pakistan cannot be fully captured into one study. Furthermore, the interview sample was purposive and only considered generations that could provide multifaceted point of view. This could potentially limit the nuanced insights one might get from engaging with elder generations.

The basis of this study was to simply provide a foundation, for research on family language practices and heritage language transmission in Pakistan to be considered and conducted at length.

The recommendations and suggestions driven from this study come in multiple steps. For families, it is recommended to consciously integrate heritage languages within daily routines, and build empathetic and informed interactions with heritage languages—stripping away shame-based language use to replace with mindful and confident engagement. Encouraging the use of heritage languages through music, shared activities and storytelling etc. is also vital toward building positive experiences with heritage languages.

For policymakers and educators, it is recommended that educational curriculums, courses and activities must include some part of heritage language practices or spaces to ensure the educational pressure to engage solely in dominant languages is reduced while a learning space is provided. Another vital step would be to develop community-based learning initiatives that bring together people through shared experiences.

For fellow researchers and academics, future research should explore heritage languages in detail and account for different ethnic groups and communities in greater detail. Comparative studies across other regions of South Asia and multiple other contexts, for instance, diaspora, colonial mindsets etc. must also be considered to identify both global as well as local challenges in heritage language maintenance. Furthermore, future research should also explore the involvement of digital media and other platforms in language use, language socialization and language maintenance across generations. One study that could potentially immortalize and broaden the role of elder generations is conducting longitudinal, intervention-based studies involving elder and younger generations in controlled spaces to learn and preserve heritage languages.

The preservation and transmission of heritage languages are not merely linguistic concerns but are deeply tied to cultural and ethnic identity, emotional wellbeing, and societal interconnectedness. As the world continues to change, proactive efforts are needed from families, educators, policymakers, and media developers to ensure these languages thrive for future generations. This study provides foundational insights and emphasizes the need for collective responsibility in sustaining linguistic and cultural heritage.

## Data Availability

The raw data supporting the conclusions of this article will be made available by the authors without undue reservation.
