# Supplementary material for: Family language policy and heritage language transmission in Pakistan—the intersection of family dynamics, ethnic identity and cultural practices on language proficiency and maintenance
Source: Front Psychol. 2025 Mar 6;16:1560755. doi: 10.3389/fpsyg.2025.1560755 (PMC11922832; doi:10.3389/fpsyg.2025.1560755)
Supplement: Supplementary file 1 [file Supplementary_file_1.docx]

**Appendix**

Table 1: Survey Questionnaire, Items & Options

| # | | Item | Question | Options (if applicable) |
| --- | --- | --- | --- | --- |
| DEMOGRAPHIC OVERVIEW | | | | |
| 1. | | **Age** | Please indicate your age. | 1. Under 18 2. 18 to 24 3. 25 to 34 4. 35 to 44 5. 45 to 55 6. 55 to 65 7. 65+ |
| 2. | | **Gender** | Please indicate your gender. | 1. Male 2. Female 3. Non-binary |
| 3. | | **Ethnicity** | Please specify your ethnic group. | 1. Punjabi 2. Sindhi 3. Pakhtun/Pashtun 4. Baloch 5. Saraiki 6. Muhajir 7. Hazara/Hazarvi 8. Kashmiri 9. Chitrali 10. Kalash 11. Wakhi 12. Balti 13. Kohistani 14. Brahui 15. Burusho (Burushaski speakers) 16. Gujarati 17. Memons 18. Makrani 19. Parsi (Zoroastrian community) 20. Marwari 21. Koli 22. Bhil 23. Siddi 24. Afghan refugees (Dari and Pashto speakers) 25. Omrui-speaking communities 26. Other (please specify) |
| 4. | | **Primary/Dominant Language** | Please specify your primary/dominant language. Primary or dominant language is the language a person uses most frequently, is most fluent in, and is most comfortable using. | - |
| 5. | | **Heritage Language** | Please specify your heritage language. A heritage language is a language that is connected to a person's cultural or ancestral roots, and is often different from the dominant language in their society. | - |
| 6. | | **Household Structure** | Please indicate what best defines your household structure. | 1. I live in a multigenerational household currently 2. I have lived in a multigenerational household before, not at present 3. I have never lived in a multigenerational household |
| 7. | | **Generational Family Role** | Please indicate your generational role in your household. | 1. Elder generation (e.g., grandparent or similar). 2. Parent generation (e.g., parent, guardian, or similar). 3. Younger generation (e.g., child, grandchild, or similar). |
| LANGUAGE PRACTICES | | | | |
| 8. | | **Self-Reported Primary Language Competence** | Rate your proficiency in your dominant/primary language. | 1. Scenarios: Reading / Writing / Speaking / Listening 2. Scale: 1 (Not at all proficient) to 5 (Highly proficient) |
| 9. | | **Self-Reported Heritage Language Competence** | Rate your proficiency in your heritage language. | 1. Scenarios: Reading / Writing / Speaking / Listening 2. Scale: 1 (Not at all proficient) to 5 (Highly proficient) |
| 10. | | **Language Use in Intergenerational Communication** | Which language(s) do you use most often in the following intergenerational contexts?   1. Communicating with the older generation (grandparents, great-grandparents) 2. Communicating with parents 3. Communicating with the younger generation (children, grandchildren) | 1. Primary Language 2. Heritage Language 3. Mix of Both 4. Other |
| 11. | | **Language Use in Different Contexts** | How frequently do you use your heritage language in the following scenarios?   1. Speaking with grandparents 2. Speaking with parents 3. Speaking with colleagues 4. Speaking with friends 5. At family gatherings 6. During cultural or religious event | 1. 1 = Never 2. 2 = Rarely 3. 3 = Sometimes 4. 4 = Often 5. 5 = Always |
| 12. | | **Code-Switching Frequency** | How often do you switch between your heritage language and dominant language in  conversations? | 1. 1 = Never  2. 2 = Rarely  3. 3 = Sometimes  4. 4 = Often  5. 5 = Always |
| ETHNIC IDENTITY | | | | |
| 13. | | **Ethnic Identity Scale/Ethnic Identity Attachment** | Questions (Agree/Disagree format)   1. I feel a strong attachment to my ethnic group. 2. Participating in cultural practices is an important part of my life. 3. Speaking my heritage language makes me feel closer to my ethnic roots. | 1. Agree = 1 2. Disagree = 0 |
| 14. | | **Cultural Identity & Language Socialization/Participation in Cultural Practices** | To what extent do family traditions and cultural events involve the use of heritage  language? | 1. 1 = Never 2. 2 = Rarely 3. 3 = Sometimes 4. 4 = Often 5. 5 = Always |
| 15. | | **Perceived Role of Heritage Language in Cultural Identity** | Do you feel speaking your heritage language strengthens your connection to your cultural  identity? | 1. 1 = Strongly disagree 2. 2 = Disagree 3. 3 = Neither agree, nor disagree 4. 4 = Agree 5. 5 = Strongly agree |
| FAMILY DYNAMICS | | | | |
| 16. | | **Self-Reported Family Language Influence** | Extended family plays a role in maintaining your heritage language. | 1. 1 = Strongly disagree 2. 2 = Disagree 3. 3 = Neither agree, nor disagree 4. 4 = Agree 5. 5 = Strongly agree |
| 17. | | **Language Practices of Grandparents** | How often do grandparents or great-grandparents speak the heritage language to younger  family members? | 1. 1 = Never 2. 2 = Rarely 3. 3 = Sometimes 4. 4 = Often 5. 5 = Always |
| 18. | | **Role of Extended Family** | To what extent do extended family members (e.g., aunts, uncles, grandparents) encourage the use of the heritage language in your household? | 1. 1 = Never 2. 2 = Rarely 3. 3 = Sometimes 4. 4 = Often 5. 5 = Always |
| 19. | | **Extended Family’s Influence on Language Decisions** | Do extended family members influence decisions about language use within your household? | 1. Yes 2. No 3. Not Sure |
| 20. | | **Parental Strategies in Language Transmission** | Which strategies do you use to ensure your children maintain the heritage language?   1. Regular practice at home 2. Cultural storytelling 3. Religious practices | 1. Selected = 1 2. Not selected = 0 |
| ATTITUDES & PERCEPTIONS | | | | |
| 21. | **Self-reported Importance of Heritage Language** | | How important is it to you that your heritage language is maintained in your family? | 1. 1 = Not important at all 2. 2 = Somewhat Important 3. 3 = Important 4. 4 = Very Important 5. 5 = Extremely important |
| 22. | **Self-reported Challenges of Heritage Language Maintenance** | | How challenging is it to maintain your heritage language in your family? | 1. 1 = Not important at all 2. 2 = Somewhat Important 3. 3 = Important 4. 4 = Very Important 5. 5 = Extremely important |
| 23. | **Preferred Language for Household Communication** | | What is the preferred language for communication within your household? | 1. 1 = Primary Language 2. 2 = Heritage Language 3. 3 = Mix of Both |
| 24. | **Attitudes Towards Bilingualism** | | 1. Being bilingual is an important skill for children in today’s world. 2. Heritage language skills will benefit my children professionally or academically | 1. Agree = 1 2. Disagree = 0 |
| 25. | **Perceived Effort & Rewards** | | How much effort do you feel is required to maintain the heritage language in your family? | 1. 1 = Very little 2. 2 = Little 3. 3 = Regular amount of effort 4. 4 = Some effort 5. 5 = A lot |
